# Optimal mean arterial pressure for favorable neurological outcomes in patients after cardiac arrest

**DOI:** 10.1186/s40560-025-00814-x

**Published:** 2025-07-31

**Authors:** Sijin Lee, Kwang-Sig Lee, Kap Su Han, Juhyun Song, Sung Woo Lee, Su Jin Kim

**Affiliations:** 1 Department of Emergency Medicine, Korea University College of Medicine & Anam Hospital, 73, Goryeodae-Ro, Seongbuk-Gu, Seoul, Republic of Korea; 2 AI Center, Korea University College of Medicine, Seoul, Republic of Korea

**Keywords:** Cardiac arrest, Post-cardiac arrest care, Mean arterial pressure, Neurological outcomes, Explainable machine learning

## Abstract

**Background:**

Optimal mean arterial pressure (MAP) range after cardiac arrest remains uncertain. This study aimed to investigate the association between MAP and neurological outcomes during the early post-resuscitation period, with the goal of identifying optimal MAP range associated with favorable outcomes.

**Methods:**

This retrospective observational study included 291 post-cardiac arrest patients treated at a tertiary care center. Five machine learning models to predict favorable neurological outcomes using hourly MAP measurements during the first 24 h after return of spontaneous circulation (ROSC) were compared and Random Forest model was selected due to its superior performance. Variable importance and Shapley Additive exPlanations (SHAP) were used to investigate the association between MAP and favorable neurological outcomes. SHAP dependence plots were used to identify optimal MAP ranges associated with favorable outcomes. In addition, individual-level predictions were interpreted using local interpretable model-agnostic explanations (LIME) and SHAP force plots.

**Results:**

Machine learning analysis showed that MAP were associated with favorable neurological outcomes, with higher variable importance during the first 6 h after ROSC. SHAP analysis revealed an inverted U-shaped relationship between MAP and favorable neurological outcomes, with an optimal threshold of 79.56 mmHg (IQR: 73.70–82.54). This threshold remained consistent across both early (1–6 h: 79.26 mmHg) and later (7–24 h: 80.09 mmHg) hours. Individual-level explanations using SHAP and LIME highlighted that maintaining higher MAP during the early post-resuscitation period contributed positively to outcome predictions.

**Conclusions:**

Machine learning analysis identified MAP as a major predictor of favorable neurological outcomes, with higher variable importance during the first 6 h after ROSC. MAP showed an inverted U-shaped relationship with favorable neurological outcomes, with an optimal threshold of approximately 80 mmHg.

**Supplementary Information:**

The online version contains supplementary material available at 10.1186/s40560-025-00814-x.

## Background

Cardiac arrest remains a major global health burden, with high mortality rates despite advances in resuscitation science [1, 2]. Although survival has improved, brain injury continues to be a leading cause of long-term disability and death among survivors [3, 4]. This injury primarily results from cerebral hypoxia during arrest and may be exacerbated by ongoing pathophysiological processes in the early post-resuscitation period [5–7]. A central component of this process is cerebrovascular dysfunction, characterized by endothelial injury and impaired vascular reactivity [7, 8]. These changes often lead to a loss of cerebral autoregulation, rendering cerebral perfusion pressure highly dependent on mean arterial pressure (MAP) [9, 10]. In this context, post-resuscitation hypotension becomes particularly detrimental, highlighting the critical need for effective blood pressure management during the early post-resuscitation period.

Current guidelines from the American Heart Association (AHA) recommend maintaining MAP above 65 mmHg in post-resuscitation care [11]. However, emerging evidence suggests that this threshold may be inadequate for ensuring sufficient cerebral perfusion [7, 12]. Several observational studies have reported that higher MAP levels during the first 24 h after the return of spontaneous circulation (ROSC), commonly referred to as the early post-resuscitation period, are associated with improved neurological outcomes [13–16]. Notably, Roberts et al. and Kilgannon et al. found that elevated MAP during the first 6 h after ROSC was independently associated with favorable neurological outcomes [13, 14]. Similarly, a systematic review by Bhate et al. concluded that MAP targets in the range of 80–100 mmHg were consistently associated with better neurologic outcomes compared to lower targets [16].

While conventional statistical methods, such as linear regression and logistic models, have contributed to understanding the relationship between MAP and neurologic outcomes, they are limited in capturing non-linear relationships and interactions among variables. In contrast, machine learning (ML) techniques can identify complex, non-linear patterns in high-dimensional clinical data without requiring predefined assumptions [17, 18]. ML methods have shown promise in enhancing risk stratification and outcome prediction in critical care by uncovering intricate relationships between predictors and outcomes [19, 20]. Moreover, recent advances in explainable ML have improved the transparency and interpretability of model outputs, addressing prior concerns about the clinical applicability of these approaches [21–23].

In this study, we applied ML techniques to investigate the association between MAP and neurological outcomes during the early post-resuscitation period, with the goal of identifying optimal MAP range associated with favorable outcomes. We further employed explainable ML methods to enhance model transparency and support clinical interpretation.

## Methods

### Ethical approval

The study protocol was approved by the Institutional Review Board of Anam Hospital (IRB No. 2023AN0104) and was conducted in accordance with the local legislation and institutional requirements. The requirement for obtaining informed consent was waived owing to the retrospective nature of the study.

### Study population

**Fig. 1:**
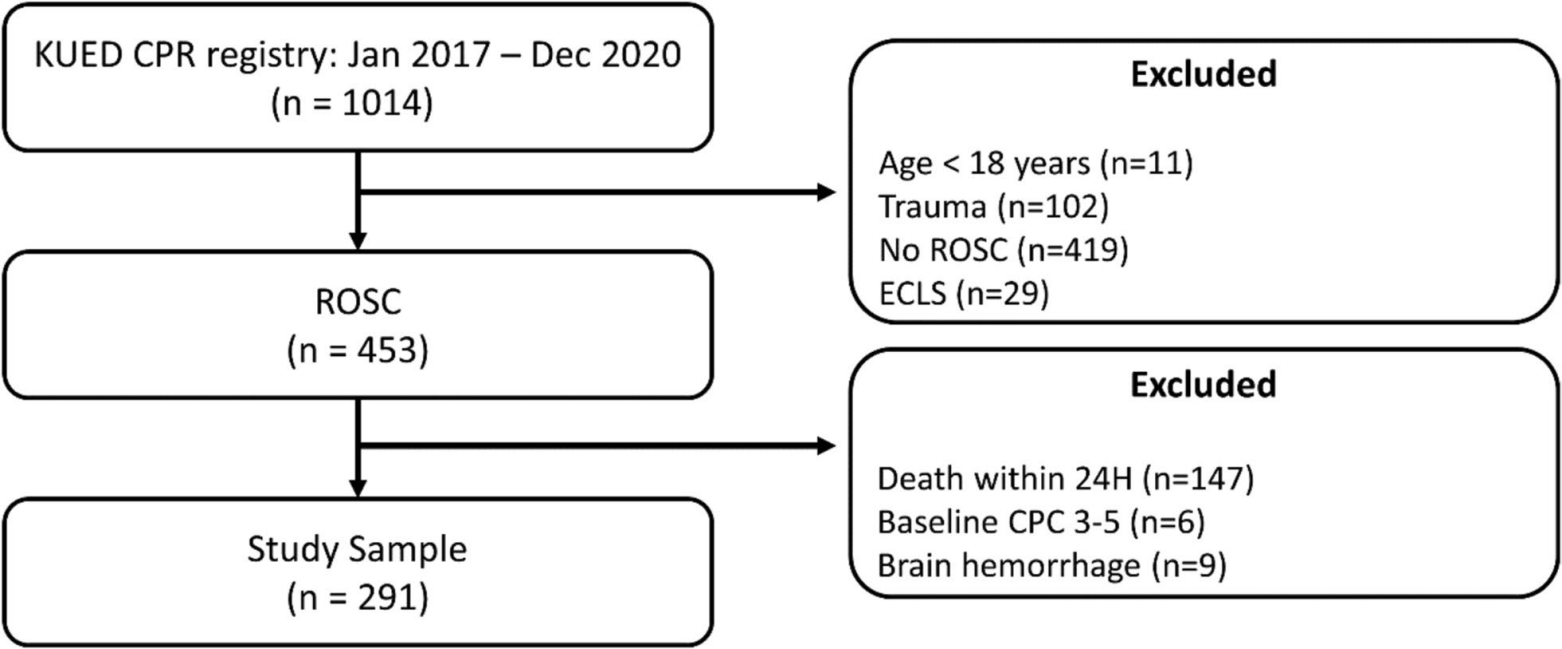
Study population. *KUED* Korea University Emergency Department, *CPR* cardiopulmonary resuscitation, *ROSC* return of spontaneous circulation, *ECLS* extracorporeal life support, *CPC* cerebral performance category

This retrospective observational study was conducted at Korea University Anam Hospital, a tertiary care center. From January 2017 to December 2020, we screened 1,014 adult cardiac arrest patients who visited the emergency department (ED). Of these, 291 patients were included in the final analysis based on the following criteria: (1) age ≥ 18 years; (2) independent functional status before cardiac arrest; and (3) Glasgow Coma Scale (GCS) score < 8 after sustained ROSC, defined as no chest compressions for 20 consecutive minutes. To ensure broad applicability, we included both in- and out-of-hospital cardiac arrests; all in-hospital cardiac arrests occurred at our own institution. Exclusion criteria were: (1) cardiac arrest of traumatic origin; (2) suspected or confirmed intracranial bleeding or stroke; (3) death within 24 h; and (4) application of mechanical circulatory support devices (e.g., extracorporeal membrane oxygenation, intra-aortic balloon pump, or Impella) during cardiopulmonary resuscitation (CPR) or after ROSC. We excluded patients who died within 24 h to ensure data reliability and maintain a homogeneous study population. A complete list of all inclusion and exclusion criteria is provided in Fig. 1.

### Post-cardiac arrest care protocols

Our institution follows a standardized, hospital-wide post-cardiac arrest care protocol based on the most recent AHA guidelines. Targeted temperature management (TTM) was applied to all eligible comatose patients following out-of-hospital or in-hospital cardiac arrest, with clinicians selecting a target temperature between 32 °C and 36 °C. The target was maintained for 24 h, followed by controlled rewarming at a rate of 0.25 °C per hour. For blood pressure management, institutional guidelines recommend maintaining a minimum MAP of ≥ 65 mmHg. While individual clinicians could set higher targets based on clinical judgment, this value served as the standardized lower limit. Norepinephrine was used as the first-line vasopressor for persistent hypotension after adequate fluid resuscitation. If MAP ≥ 65 mmHg could not be achieved with moderate doses, additional agents—such as vasopressin, epinephrine, dopamine, dobutamine, or milrinone—were considered according to the patient’s hemodynamic profile and presumed etiology of arrest. Neurological prognostication was deferred until at least 72 h after normothermia in patients receiving TTM, or 72 h after ROSC in those not cooled. A multimodal prognostication protocol was followed, including daily clinical examinations (pupillary and corneal reflexes, motor response, myoclonus), amplitude-integrated EEG monitoring for 3–5 days, serial neuron-specific enolase (NSE) measurements, and diffusion-weighted MRI between days 2 and 7 to assess anoxic brain injury.

### Data collection and management

The dependent variable was binary neurological outcome measured by the cerebral performance categories (CPC) scale, which emergency medical services (EMS) personnel assessed and recorded in the registry at discharge: scores 1–2 (favorable) vs. 3–5 (unfavorable, e.g., severe brain injury or death) [24]. Based on clinical relevance in resuscitation practice from existing literature [24, 25], 46 independent variables were selected across four categories: demographic (age, sex, comorbidities), cardiac arrest-related factors (out-of-hospital cardiac arrest, bystander CPR, witnessed arrest, CPR duration (time from CPR start to sustained ROSC), cardiac causes, shockable rhythm), treatment-related factors (percutaneous coronary intervention (PCI), vasoactive-inotropic score (VIS), targeted temperature management (TTM)), GCS score after ROSC (GCS_ROSC), and hourly MAP measurements in initial 24 h post-ROSC. PCI was included only if performed within the first 24 h after ROSC, to reflect early post-resuscitation management. TTM was included due to its potential influence on neuroprotection and hemodynamic management [26].

All patients had an arterial line placed either during resuscitation or immediately after ROSC for blood pressure monitoring. In cases where early arterial line placement was challenging or data were missing, which accounted for 2.3% of all MAP data, MAP was estimated using non-invasive systolic and diastolic blood pressure measurements. These were calculated by adding one-third of the systolic blood pressure to the diastolic pressure. We analyzed hourly MAP data collected during the first 24 h after ROSC, as this period represents a critical therapeutic window for neurological recovery [8]. Prior studies have similarly focused on this early phase, demonstrating that blood pressure patterns during the first 24 h are strongly associated with mortality and neurological outcomes in post-cardiac arrest patients [27, 28].

The VIS was calculated hourly by quantifying the doses of vasopressors administered each hour using the following formula [29]:

VIS = dopamine dose (μg/kg/min) + dobutamine dose (μg/kg/min) + 100 × epinephrine dose (μg/kg/min) + 10 × milrinone dose (μg/kg/min) + 10,000 × vasopressin dose (unit/kg/min) + 100 × norepinephrine dose (μg/kg/min).

We included two VIS-related variables in our machine learning model: the total sum of VIS over the 24-h period (VIS_total) and the highest hourly VIS value during the 24 h (VIS_max). Including both variables allowed us to capture both the cumulative vasopressor exposure and the peak vasopressor requirement. Approximately 5% of the hourly VIS were missing due to intermittent documentation lapses or transmission delays from bedside monitors. These missing data points were temporally isolated and showed no systematic pattern, supporting a classification of missing at random. To maintain the continuity of the physiological time series without introducing bias, missing VIS values were imputed using simple linear interpolation between adjacent non-missing timepoints.

### Statistical analyses

We compared five machine learning models to predict favorable neurological outcomes, including Random Forest, Decision Tree, Naive Bayes, Support Vector Machine, and Logistic Regression. Among these, the Random Forest (RF) model showed superior performance and was selected as our primary model. The RF model comprised 100 decision trees, with the data split into training and validation sets at an 80:20 ratio (233 vs. 58 cases). We calculated variable importance in the RF model based on the average decrease in node impurity (Gini impurity) across all trees when splits were made on each variable. Major predictors were defined as variables ranked in the top 20 among all variables in the prediction model. Furthermore, to enhance the interpretability of the RF model and address the black-box issue, we utilized visualized explanation at variable and individual levels. At the variable level, the Shapley Additive exPlanation (SHAP) summary plot was used to illustrate the strength as well as the direction of associations between major predictors and favorable neurological outcomes, and SHAP dependence plots were used to identify the optimal MAP ranges associated with favorable neurological outcomes [30–32]. For further explanation at the individual level, we used local interpretable model-agnostic explanations (LIME) and SHAP force plots to explain how specific variables contributed to individual predictions. LIME approximated the classifier with a locally linear model to elucidate the contributions of variables such as MAP. SHAP force plots visualized the cumulative impact of these variables, showing how positive and negative contributors affected the likelihood of favorable neurological outcome. These interpretable techniques enhanced the reliability and transparency of the ML models for clinical applications [33–35]. All analyses were performed using Python version 3.8.17.

## Results

Of the 291 patients included in the study, 109 (37.5%) achieved favorable neurological outcomes, while 182 (62.5%) had unfavorable outcomes. Baseline characteristics and clinical factors differed significantly between the two groups (Table 1). Patients with favorable outcomes had a higher incidence of witnessed cardiac arrest (91.7% vs. 79.1%, *p* = 0.006), cardiac causes (56.0% vs. 20.3%, *p* = 0.027), and initial shockable rhythms (79.8% vs. 13.2%, *p* < 0.001). This group also demonstrated shorter CPR duration (median 6 min [IQR: 3–9] vs. 23 min [IQR: 9–34], *p* < 0.001), a higher rate of PCI (37.6% vs. 8.8%, *p* < 0.001), and lower VIS_max (median 21.32 μg/kg/min [IQR: 8.71–48.41] vs. 53.33 μg/kg/min [IQR: 23.81–53.33], *p* = 0.001).

**Table 1:** Baseline characteristics of the study population and comparison between patients with favorable and unfavorable neurological outcomes

	All (*n* = 291)	Favorable (*n* = 109)	Unfavorable (*n* = 182)	*p* value
Age, years	65 (56–75)	63 (55–70)	68 (56–77)	0.008
Male, *n* (%)	189 (64.9)	71 (65.1)	118 (64.8)	0.958
Comorbidities				
Hypertension, *n* (%)	122 (41.9)	46 (42.2)	76 (41.8)	0.941
Heart failure, *n* (%)	23 (7.9)	7 (6.4)	16 (8.8)	0.468
Coronary artery disease, *n* (%)	46 (15.8)	19 (17.4)	27 (14.8)	0.557
Neurological Disease, *n* (%)	31 (10.7)	7 (6.4)	24 (13.2)	0.070
COPD/Asthma, *n* (%)	28 (9.6)	5 (4.6)	23 (12.6)	0.024
Liver cirrhosis, *n* (%)	9 (3.1)	1 (0.9)	8 (4.4)	0.133
Diabetes, *n* (%)	93 (32.0)	28 (25.7)	65 (35.7)	0.076
Chronic Kidney Disease, *n* (%)	28 (9.6)	8 (7.3)	20 (11.0)	0.307
Malignancy, *n* (%)	27 (9.3)	9 (8.3)	18 (9.9)	0.642
Cardiac arrest				
Out of Hospital CA, *n* (%)	227 (78.0)	87 (79.8)	140 (76.9)	0.564
Bystander CPR, *n* (%)	227 (78.0)	91 (83.5)	136 (74.7)	0.081
Witnessed CA, *n* (%)	244 (83.8)	100 (91.7)	144 (79.1)	0.006
CPR duration^a^, min	11 (5–11)	6 (3–9)	23 (9–34)	< 0.001
Cardiac causes, *n* (%)	98 (34.6)	61 (56.0)	37 (20.3)	0.027
Shockable rhythm, *n* (%)	111 (38.1)	87 (79.8)	24 (13.2)	< 0.001
Treatment				
VIS_max^b^	21.6 (2.2–85.3)	14.2 (0.0–38.4)	37.8 (3.9–147.8)	0.002
VIS_total^c^	219.2 (8.9–789.6)	96.4 (0.0–257.3)	337.5 (16.7–803.5)	< 0.001
PCI, *n* (%)	57 (19.6)	41 (37.6)	16 (8.8)	< 0.001
TTM, *n* (%)	106 (36.4)	39 (35.8)	56 (30.8)	0.092
Mean arterial pressure				
1H	85.0 (71.0–99.2)	92.0 (81.0–101.2)	80.0 (66.0–93.2)	< 0.001
6H	84.5 (66.8–102.0)	90.0 (81.8–108.0)	73.0 (59.8–100.0)	< 0.001
12H	83.0 (72.0–95.0)	85.0 (77.0–97.2)	79.5 (69.0–91.0)	0.004
24H	82.0 (71.0–94.2)	84.5 (75.0–99.0)	79.5 (68.8–90.2)	< 0.001

*IQR* inter-quartile range, *COPD* chronic obstructive pulmonary disease, *CA* cardiac arrest, *CPR* cardiopulmonary resuscitation, *ROSC* return of spontaneous circulation, *STEMI* ST elevation myocardial infarction, *PCI* percutaneous coronary intervention, *VIS* vasoactive-inotropic score, *TTM* targeted temperature management

^a^CPR duration was defined as the time from CPR start to sustained ROSC

^b^VIS_max was defined as the highest VIS value over the first 24 h after ROSC

^c^VIS_total was defined as the total sum of VIS value over the first 24 h after ROSC

We evaluated five ML algorithms to predict favorable neurological outcomes using baseline variables and hourly MAP values obtained during the first 24 h after ROSC. Among them, the Random Forest model demonstrated the highest performance, with an accuracy of 0.907, F1-score of 0.857, and area under the curve (AUC) of 0.932. This model was selected for further analysis. Performance metrics for all models are summarized in Table 2.

**Table 2:** Model performance for predicting favorable neurological outcome in patients after cardiac arrest

	Accuracy	Precision	Recall	F1-score	AUC
Random forest	0.907	0.833	0.882	0.857	0.932
Decision tree	0.833	0.722	0.765	0.743	0.918
Naive Bayes	0.889	0.762	0.941	0.842	0.893
Support vector machine	0.852	0.800	0.706	0.750	0.926
Logistic regression	0.833	0.700	0.824	0.757	0.908

*AUC* area under the curve

**Fig. 2:**
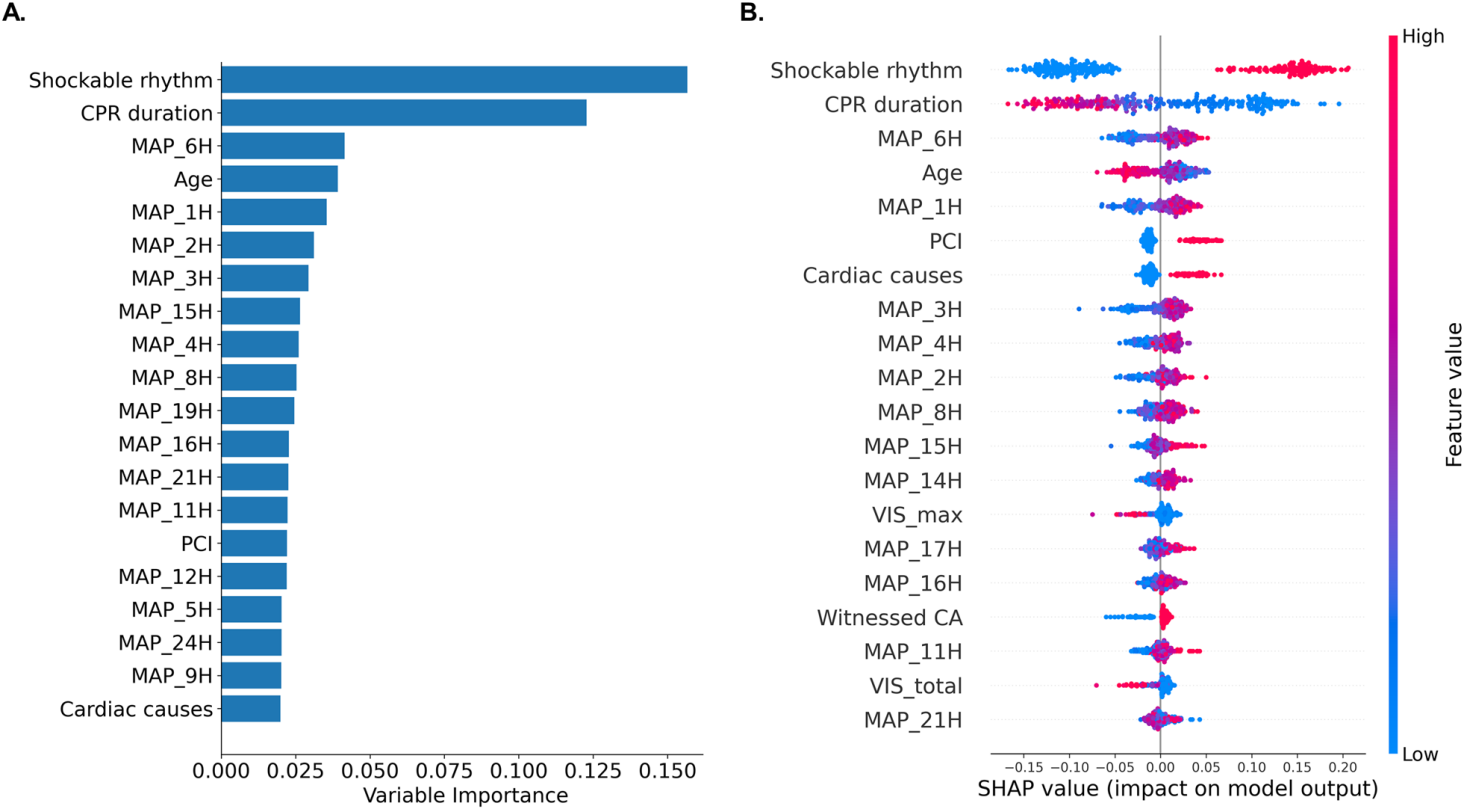
Variable importance rankings and SHAP summary plot of the Random Forest model predicting favorable neurological outcomes after cardiac arrest. **A** Bar plot indicating the variable importance of major predictors based on mean decrease in impurity. **B** SHAP summary plot illustrating the direction and magnitude of each predictor’s impact on the model output. *MAP_#H* mean arterial pressure recorded at #hour after return of spontaneous circulation, *SHAP* Shapley Additive exPlanations, *MAP* mean arterial pressure, *CPR* cardiopulmonary resuscitation, *CA* cardiac arrest

Figure 2 illustrates the variable importance rankings and SHAP summary plot derived from the Random Forest model. As shown in Fig. 2A, initial shockable rhythm and CPR duration emerged as the most influential predictors of favorable neurological outcomes. MAP during the first 6 h after ROSC (MAP_1H-6H) also ranked among the top predictors, showing greater importance than MAP values from later timepoints. The SHAP summary plot (Fig. 2B) provides a visual and quantitative depiction of each variable’s impact on model predictions. Favorable outcomes were strongly associated with initial shockable rhythms, shorter CPR durations, and younger patient age. In addition, higher MAP values during the first 6 h after ROSC were consistently associated with positive SHAP values, indicating that elevated MAP during this early period contributes positively toward favorable neurological outcomes. Regarding VIS, both VIS_max and VIS_total demonstrated comparable contributions on outcome prediction. The full ranking of all variables by importance and SHAP values is presented in Supplementary Figure S1.

**Fig. 3:**
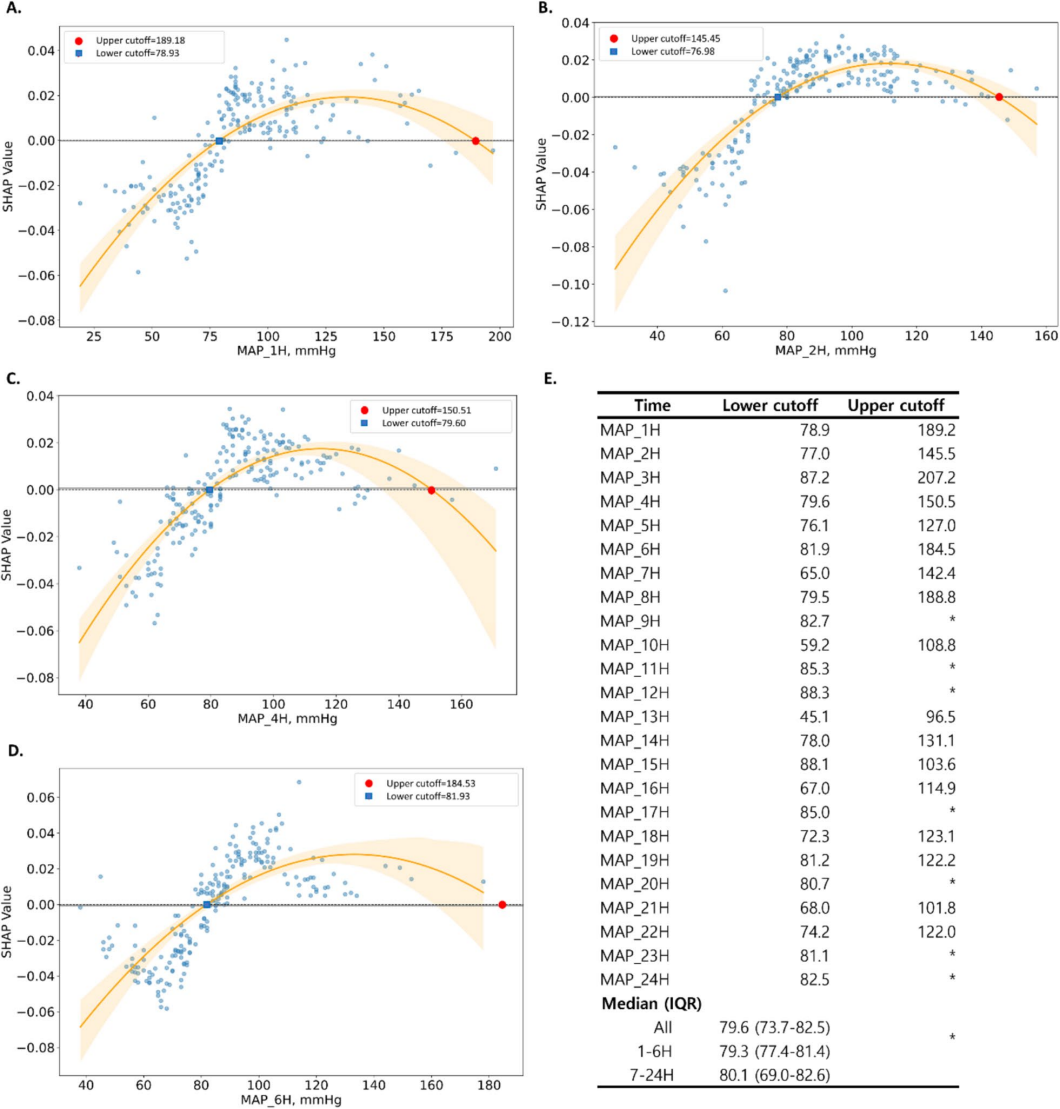
SHAP dependence plots for mean arterial pressure at different timepoints. **A**–**D** SHAP dependence plots for MAP measured at 1H, 2H, 4H, and 6H. The *x*-axis represents MAP values (mmHg), the *y*-axis represents SHAP values, the orange curve is the LOWESS fit with its 95% confidence band (shaded). SHAP values greater than zero indicate an increased probability of a favorable neurological outcome. Red and blue markers denote the lower and upper MAP cutoff values identified for each timepoint. **E** Lower and upper MAP cutoff values during the first 24 h after return of spontaneous circulation. Asterisks (*) indicate timepoints, where an upper cutoff could not be derived. *MAP_#H* mean arterial pressure recorded at #hour after return of spontaneous circulation, *SHAP* Shapley Additive exPlanations, *LOWESS* LOcally Weighted Scatterplot Smoothing

To identify optimal MAP ranges associated with favorable neurological outcomes, cutoff values were derived from SHAP dependence plots at each hourly timepoint during the first 24 h after ROSC (Fig. 3A-–D). The lower cutoff values represent MAP thresholds above which the model predicted an increased likelihood of favorable outcome. While upper cutoff values were also identified, their clinical interpretation was limited due to a small number of cases at the upper range.

Based on these SHAP-derived lower cutoff values, the median optimal MAP threshold across the 24-h period was 79.56 mmHg (IQR: 73.70–82.54) (Fig. 3E). When stratified by time, the optimal MAP threshold during the early hours (1–6 h) was 79.26 mmHg (IQR: 77.47–81.35), comparable to that of the later hours (7–24 h), 80.09 mmHg (IQR: 69.04–82.63). No statistically significant differences were observed between these time intervals, indicating consistency in optimal MAP thresholds throughout the post-resuscitation period.

**Fig. 4:**
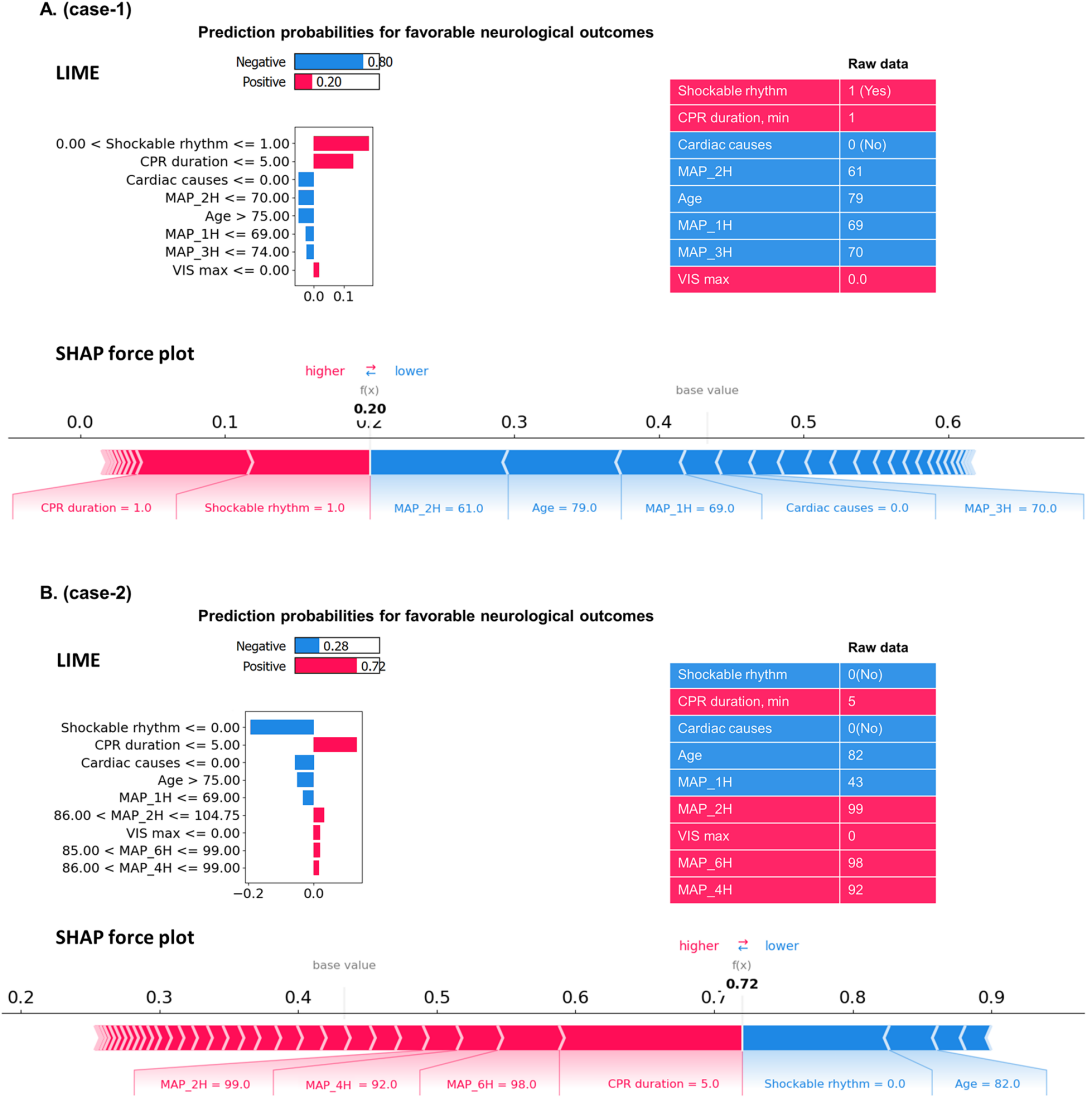
Patient-specific LIME and SHAP explanations for neurological outcome prediction. **A** Case 1—unfavorable neurological outcome (predicted probability = 0.20). **B** Case 2—favorable neurological outcome (predicted probability = 0.72). The upper panel is a LIME bar chart: features are ranked by influence, with red bars pushing the prediction toward a favorable outcome and blue bars pulling it toward an unfavorable one; the table to the right shows the patient’s raw values. The lower panel is a SHAP force plot: the gray vertical line marks the model’s baseline probability; red segments shift the prediction rightward (higher probability) and blue segments leftward (lower probability); segment width is proportional to each feature’s contribution. *MAP_#H* mean arterial pressure measured # h after return of spontaneous circulation (ROSC), *VIS* vasoactive-inotropic score, *LIME* local interpretable model-agnostic explanations, *SHAP* shapley additive explanations

To further elucidate the role of MAP at the individual level, two representative cases were analyzed using LIME and SHAP force plots (Fig. 4). In Case 1, although favorable predictors such as an initial shockable rhythm and short CPR duration (1 min) were present, the model predicted only a 20% probability of a favorable outcome, primarily due to low MAP values during the early period (MAP_1H, MAP_2H, MAP_3H).

In contrast, Case 2 involved an 82-year-old patient with a non-shockable rhythm—typically unfavorable predictors—yet the model predicted a 72% probability of a favorable outcome. This was attributed to elevated MAP values during the early period (MAP_2H, MAP_4H, MAP_6H), which appeared to offset the negative influence of other clinical factors. These findings were visually demonstrated through SHAP force plots (Fig. 4A, B), highlighting how individualized outcome predictions were shaped by the interplay of clinical variables.

## Discussion

In this study, we used ML analysis to investigate the association between MAP and favorable neurological outcomes during the early post-resuscitation period. The Random Forest model identified initial shockable rhythm and CPR duration as the most influential predictors, with MAP emerging as another major predictor. Notably, MAP values during the first 6 h after ROSC showed relatively higher importance compared to MAP values measured later hours. SHAP analysis revealed an inverted U-shaped relationship between MAP and favorable neurological outcomes, with an optimal threshold near 80 mmHg. This threshold remained consistent across both the early (1–6 h: 79.26 mmHg, IQR: 77.47–81.35) and later (7–24 h: 80.09 mmHg, IQR: 69.04–82.63) hours. These findings suggest that maintaining MAP at or above 80 mmHg throughout the post-resuscitation period may support neurological recovery.

Post-cardiac arrest brain injury evolves through a complex cascade beginning with global cerebral ischemia, followed by reperfusion and delayed secondary injury [5, 6]. The reperfusion phase is marked by brief hyperemia, then a latent period in which partial metabolic recovery occurs, though vulnerability to injury persists. This is followed by a secondary deterioration phase, characterized by cytotoxic edema, impaired oxidative metabolism, and progressive neuronal damage. This progressive deterioration highlights the importance of early hemodynamic stabilization to prevent irreversible brain injury. Prior studies support this concept: Roberts et al. found that elevated MAP within the first 6 h post-ROSC was associated with improved neurological outcomes [13], and Kilgannon et al. showed a time-weighted average MAP above 70 mmHg within 6 h after ROSC was linked to better outcomes [14]. Our findings are consistent with this evidence, reinforcing the need to optimize hemodynamic targets early in post-resuscitation care.

Our results also align with other observational studies emphasizing the benefit of higher MAP targets. A recent systematic review highlighted the existence of individualized optimal MAP (MAPOPT) values and the potential benefits of higher MAP targets after cardiac arrest [36, 37]. Notably, while previous studies estimated MAPOPT using physiological indicators, such as transcranial Doppler and pressure reactivity index, methods with limited interpretability and applicability, this study derived patient-specific optimal MAP thresholds directly from clinical data using explainable machine learning models, enhancing clinical relevance. In addition, the MAPOPT values reported in prior literature were generally above 70 mmHg, and our observed optimal MAP near 80 mmHg provides a quantitative affirmation of this trend. The optimal MAP target in this study is also consistent with recent recommendations suggesting a target MAP of 80 mmHg in settings, where advanced cerebral monitoring is not available [38].

Similarly, previous retrospective studies have shown that prolonged exposure to MAP values below 60 mmHg during the first 24 h after ROSC was independently associated with increased ICU mortality in post-cardiac arrest patients [28]. These findings highlight the clinical importance of avoiding sustained hypotension, reinforcing our conclusion that maintaining MAP at or above 80 mmHg may help improve neurological and overall outcomes. In addition, a large retrospective study using national ICU registry data found that both low and high MAP values during the first 24 h after ROSC were associated with increased hospital mortality, with the lowest mortality observed at MAP levels around 60–63 mmHg [27]. The U-shaped relationship in the study aligns with our finding of an optimal MAP threshold near 80 mmHg and reinforces the finding that both hypotension and excessive hypertension are harmful in post-cardiac arrest care.

Despite agreement with observational data, our results differ from several randomized trials that reported no significant benefit of higher MAP targets in post-cardiac arrest care. Kjaergaard et al. found no difference in neurological outcomes between patients randomized to MAP targets of 63 mmHg and 77 mmHg [39]. Other trials similarly found no differences in neurological outcomes or neuron-specific enolase levels across MAP target groups [40, 41]. However, these studies had several limitations: initial interventions were delayed (median 146–190 min post-ROSC), the cohorts were narrow (86–96% with initial shockable rhythms), and the difference in achieved MAP between groups was modest (10.7 mmHg, 95% CI 10.0–11.4). In particular, the time required for randomization likely delayed blood pressure management. Given that the first 6 h after ROSC were considered as a critical therapeutic window, this delay may have attenuated the potential benefits of MAP management in those trials.

While ML methods are increasingly used in healthcare, their application in post-cardiac arrest care remains limited—largely due to concerns about interpretability. In high-stakes environments like critical care, clinicians are unlikely to act on model predictions without a clear understanding of the rationale. Explainable ML aims to address this barrier by providing transparent, clinically meaningful insights. In this study, we used SHAP and LIME to interpret how specific MAP values contributed to outcome predictions. SHAP, in particular, is well-suited for explaining predictions in tree-based models such as Random Forests and is increasingly adopted in clinical ML research. Future studies should build on this approach by examining interaction effects between variables and applying dynamic modeling frameworks. The overarching goal is not to reveal every aspect of the ML algorithm but to provide interpretable, actionable guidance for clinical decision-making.

This study has several limitations. As a retrospective analysis, it is subject to potential confounding and cannot establish causality between MAP and neurological outcomes. We also lacked long-term follow-up data beyond hospital discharge, limiting our ability to assess sustained functional recovery. Moreover, although MAP is widely used as a surrogate for cerebral perfusion, it remains an indirect marker and may not fully reflect individual variability in cerebral blood flow or autoregulation. Furthermore, the limited influence of GCS_ROSC likely reflects its narrow distribution within our cohort, as the majority of patients were deeply comatose at enrollment with GCS scores clustering around 3. These limitations underscore the need for prospective, multicenter randomized controlled trials to determine optimal hemodynamic strategies in post-cardiac arrest care.

## Conclusions

ML analysis identified MAP as a major predictor of neurological outcomes, with relatively high importance during the first 6 h after ROSC. An inverted U-shaped relationship was observed between MAP and favorable neurological outcomes, with an optimal threshold of approximately 80 mmHg. This threshold remained consistent during the early post-resuscitation period. These findings suggest that maintaining MAP at or above 80 mmHg may support neurological recovery following cardiac arrest.

## Supplementary Information

Additional file 1.

## Data Availability

The data sets used and/or analyzed during the current study are available from the corresponding author on reasonable request.

## Abbreviations

MAP: Mean arterial pressure
AHA: American heart association
ICU: Intensive care unit
ED: Emergency department
IRB: Institutional review board
GCS: Glasgow Coma Scale
ROSC: Return of spontaneous circulation
TTM: Targeted temperature management
CPR: Cardiopulmonary resuscitation
EMR: Electronic medical record
VIS: Vasoactive-inotropic score
PCI: Percutaneous coronary intervention
AUC: Area under the curve
SHAP: Shapley additive exPlanantions
CPC: Cerebral performance category
NSE: Neuron-specific enolase
