# Supplementary material for: Optimal mean arterial pressure for favorable neurological outcomes in patients after cardiac arrest
Source: J Intensive Care. 2025 Jul 31;13:42. doi: 10.1186/s40560-025-00814-x (PMC12315296; doi:10.1186/s40560-025-00814-x)
Supplement: Supplementary file 1 — Additional file 1. [file 40560_2025_814_MOESM1_ESM.docx]

**Supplementary Information**


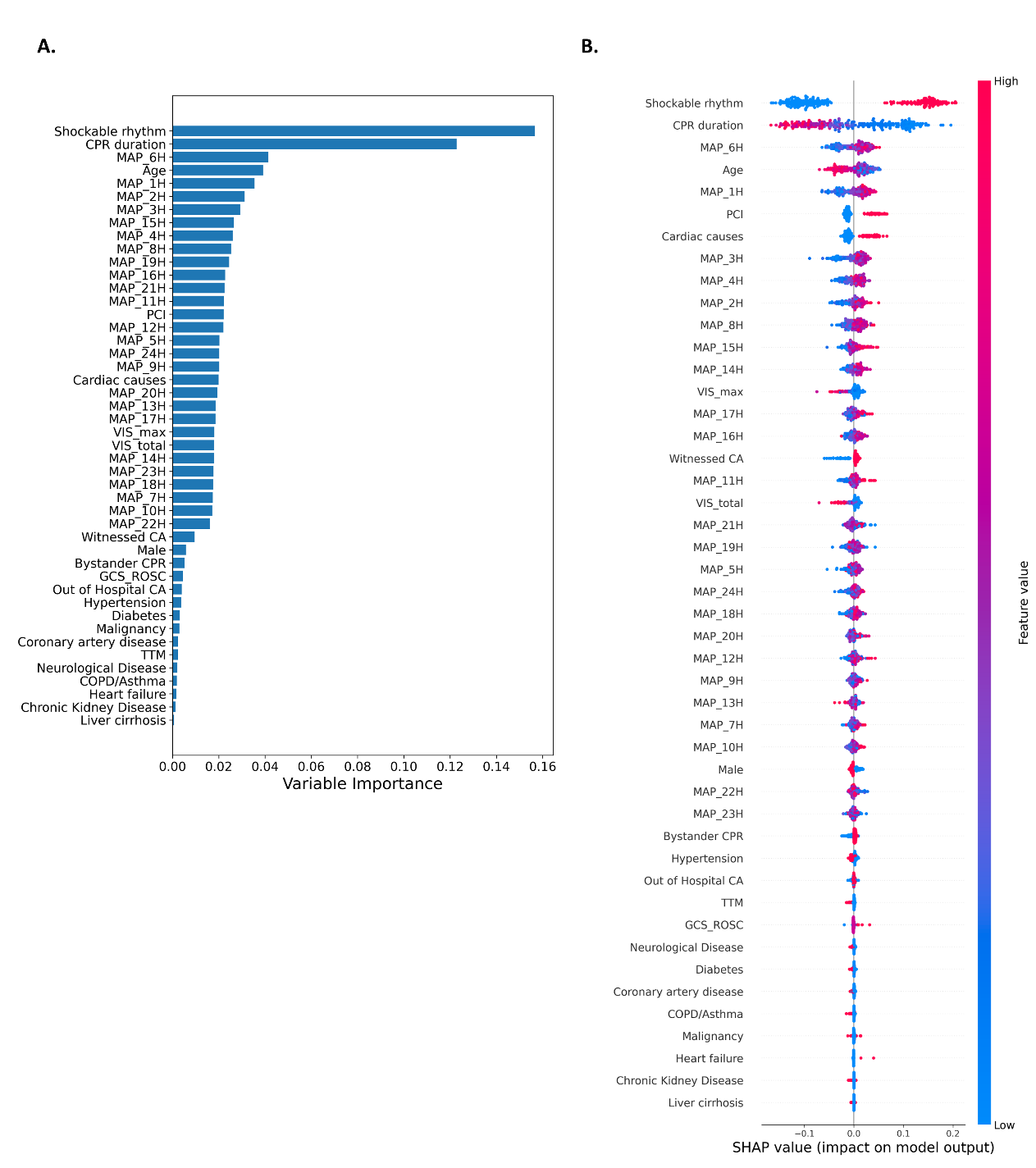
**Supplementary Figure S1. Variable importance rankings and SHAP summary plot of the Random Forest model predicting favorable neurological outcomes after cardiac arrest. A.** Bar plot indicating the variable importance of predictors based on mean decrease in impurity. **B.** SHAP summary plot illustrating the direction and magnitude of each predictor’s impact on the model output. MAP_#H, mean arterial pressure recorded at #hour after return of spontaneous circulation; SHAP, Shapley Additive exPlanations; MAP, mean arterial pressure; CPR, cardiopulmonary resuscitation; CA, cardiac arrest; GCS_ROSC, Glasgow Coma Scale after return of spontaneous circulation.
